# Thoracoscopic giant lung bullaectomy: our initial experience

**DOI:** 10.1186/s13019-022-01780-3

**Published:** 2022-03-15

**Authors:** Marina Kolodii, Sharbel Azzam, Michael Peer

**Affiliations:** Department of Thoracic Surgery, Tel Aviv Medical Center, Affiliated with Sackler School of Medicine, Tel-Aviv University, 6 Weizman Street, Tel Aviv, Israel

**Keywords:** Giant lung bullae, Thoracoscopy, Bullectomy, Thoracotomy, Lung emphysema

## Abstract

**Background:**

Giant lung bullae (GLB) are rare, and the only currently available management involves either an open surgical resection (thoracotomy) or the newer minimally invasive resection consisting of video-assisted thoracoscopic surgery (VATS). The aim of our study was to evaluate the possible influence of GLBs pulmonary attachment on patient's post-operative complications.

**Methods:**

A retrospective analysis included all consecutive patients with GLBs who underwent bullae's surgical resection from 7/2007 to 12/2018. GLBs patient's individual characteristics, including demographics, comorbidities, and clinical pre-operative, surgical intra-operative and post-operative data were evaluated.

**Results:**

20 patients with GLBs, 15 males and 5 females with average age of 48.9 years (range, 22–67 years) underwent 21 surgical procedures. The GLBs were located in the right lung in 12 patients, in the left lung in seven patients, and in both lungs in one patient. Fifteen patients (75%) were symptomatic on admission and underwent urgent surgery. Five asymptomatic patients (25%) were operated on electively. Thirteen from 21 surgical procedures (61.9%) were VATS bullectomy, while the other eight were thoracotomies (38.1%). Complications included pneumonia successfully treated with intravenous antibacterial therapy in two thoracotomy patients and in one VATS patient (three patients, 14.2%) and a prolonged air leak in two thoracotomy and four VATS patients (six patients, 28.5%). Out of 21 GLBs, eight had a wide attachment with lung parenchyma (wide-based bullae's) and 13 had a short attachment (short-based bullae's). Two re-operated patients, with prolonged air leak complicated with empyema, had a wide-based GLBs. The median hospital stay was nine days. All patients completed the 24-month follow-up.

**Conclusions:**

Minimally invasive video-assisted thoracoscopic surgery as an open thoracotomy surgery is a safe and effective for giant lung bullae (GLB). Patients with wide-based GLBs were more likely to develop postoperative prolonged air leak that requiring re-operation.

## Introduction

Bullous lung disease is a form of pulmonary tissue destruction that may be either well-defined or dispersed. Bullae are defined as air spaces in the lung larger than one centimeter in diameter. They may be single or multiple and tend to enlarge with time and can reach huge dimensions: those that occupy more than 30% of the hemithorax are called “giant bullae”. Lung bullae may be asymptomatic and identified only as an incidental finding, although they usually cause symptoms of dyspnea. Bullous formation is believed to be promoted by various factors, among them significant cigarette smoking, cocaine smoking, pulmonary sarcoidosis, 1-antitrypsin deficiency, Marfan’s syndrome, Ehlers-Danlos syndrome, and inhaled fiberglass exposure [1–3]. Although the management of causative factors is important, preventive approaches, such as smoking cessation, pulmonary rehabilitation, and treatment of alpha–1—antitrypsin deficiency and chronic obstructive pulmonary disease may slow the progress of the lung bullous disease but cannot reverse it.

Surgery is not considered the gold standard therapy for bullous lung disease [4]. In selected cases, however, it remains the only option because of the absence of any other effective treatments. The first surgical resection of bullae was reported in 1939 by Kaltreider and Fray [5]. Today, resection of bullae is usually limited to a symptomatic patient with a large bulla that occupies more than 30% of the hemithorax or in a patients with complications, such as infection, secondary pneumothorax, persistent air leak, or bleeding [1] or mediastinal shift with herniation of the bullous lung tissue to the contralateral hemithorax [6]. The factors affecting the outcome of bullectomy are not well known, and the surgical results differing from center to center [6–9]. Various surgical procedures, such as intracavitary bullous drainage, bullae resection, plication, lung volume reduction and lobectomy are traditionally performed via an open approach, such as thoracotomy or sternotomy, but they are associated with high morbidity and mortality rates. Today, minimally invasive surgery, primarily by video-assisted thoracoscopic surgery (VATS) stapling technique, considered more suitable and safe treatment option for giant lung bullae (GLB) [10, 11]. Reported, that resection of wide-based GLB in comparison to short-based GLB, is technically more difficult because of requiring large-volume resection of underling pulmonary parenchyma [12]. We goaled to review our experience with surgical treatment for all GLBs, with short-based or wide-based pulmonary attachment, and analyze the associated morbidity, mortality, duration of hospitalization, and complication rates.

## Methods

Surgical intervention by open or thoracoscopic techniques for removing giant lung bullaes (GLBs) should affect the postoperative course of the patients and make the decision making process for choosing the optimal surgical technique challenging. We aimed retrospectively review the surgical results and postoperative outcome of all patients with GLBs operated at the Department of Thoracic Surgery, Shamir Medical Center (formerly Assaf Harofeh), Israel, between July 2007 and December 2018. The data were collected from the clinical charts and from the surgical and pathology reports and included demographics, clinical characteristics, comorbidities, bullae location and size, bullae's pulmonary attachment, wide base or short base, side and type of surgery, surgical techniques, postoperative complications and hospital stay. Follow-up was complete for all patients (Table 1).

**Table 1:** Patient characteristics

Variable	No	%
Males	15	75
Females	5	25
Smoker	7	35
Non-smoker	8	40
Ex-smoker	5	25
Marijuana smoker	1	5
Secondary spontaneous pneumothorax	9	45
Non-resolving pneumothorax	4	20
Recurrent pneumothorax	3	15
Dyspnea	5	25
Video-assisted thoracoscopic surgery	13	61.9
Open surgery	8	38.1
Conversion surgery	1	5
Right-sided surgery	12	60
Left-sided surgery	7	35
Bilateral surgery	1	5
Single bullae	7	35
Multiple bullae	13	65
Re-thoracotomy	2	10
Elective surgery	5	25
Urgent surgery	15	75

### Subjects

We retrospectively analyzed the data of 20 patients who underwent surgical treatment for GLB (21 procedures). The patients included to the study had GLBs that occupied at least one-third of the hemithorax, had a respiratory symptoms due to raptured bullae or induced by compressing of involved by bullae lung parenchyma or contralateral lung due to mediastinal shifting.

### Ethics approval

The study was approved by the Shamir Medical Centers Institutional Ethical Committee (Approval Number: 0179-18-ASF), and all patients gave their written informed consent prior to undergoing surgery.

All patients underwent complete preoperative assessment comprised of a general blood analysis (blood count, chemistry and coagulation), imaging studies (chest x-ray and computerized tomography (CT), and cardiac evaluation (electrocardiogram). Bullous emphysema was classified preoperatively according to the De Vries and Wolfe classification [11, 13]: single large bullae with normal underlying lung (Group I), multiple large bullaes with normal underlying lung (Group II), large bullae with underlying diffuse emphysema (Group III) or multiple bullaes with other underlying lung diseases (Group IV). GLBs attachment with lung parenchyma (wide-based or short-based bullae's) were recorded intraoperatively.

Mortality was defined as “early” when it occurred during hospitalization, and “late” when it occurred during the 6-month postoperative period. Prolonged air leak was defined as an air leak that continued for more than 7 days postoperatively.

### Surgical techniques

Thoracotomy was performed by a standard posterolateral serratus-sparing thoracotomy and VATS though the next technique: the 10-mm port was placed in the eighth intercostal space in the posterior axillary line for a 10-mm 30-degree end-viewing thoracoscope; two additional working ports were placed in the sixth intercostal space in the mid-clavicle line (10 mm), and in the fourth or fifth intercostal space just beneath the latissimus dorsi muscle, according to bullae location (30 mm). Both the thoracotomy and VATS were performed with the patient under general anesthesia and double-lumen endotracheal intubation.

During the VATS techniques, the giant bullae were punctured if they were too large to enable viewing the thoracic cavity. Any adhesions between the bullae and chest wall or lung parenchyma were dissected to expose the bullae's attachment: wide-based GLB or short-based GLB. The walls of the bullae were then grasped, and the lung parenchyma beneath the giant bullae was sutured by endoscopic mechanical staplers (Johnson & Johnson, Ethicon Endo-Surgery, Medical Devices Companies Inc, USA) in 12 patients with short bullae attachment (13 surgeries, 65%, Group I), and in eight patients with diffuse bullae attachment (8 surgeries, 35%, Group II). GLBs pulmonary attachment was generally reinforced by peri-strips (Baxter, Synovis Life Technologies Inc, USA). The lung was inflated without the addition of mechanical or chemical pleurodesis.

One or two 36 French chest tubes were inserted into the pleural cavity after surgery. No suction was used unless a severe air leak was apparent. When an air leak occurred, we used continuous low motor (− 10 to − 12 cm H2O) suction with the drainage bottle system (Biometrix Inc, Netherlands). The chest tubes were removed when no air leak was detected and when the fluid drainage was less than 100 mL/day, and the lung was fully or nearly fully expanded on chest radiography after clamping the chest tube for 12–24 h.

## Results

Twenty-one operations were performed on 20 patients, all of whom completed a follow-up of 24 months. Nineteen patients had single GLBs, and one patient had bilateral GLB. Fifteen patients were males and five were females, and their average age was 48.9 years (range, 22–67 years). Seven patients were active smokers on admission, including one marihuana smoker, five patients had a history of smoking, and eight patients were nonsmokers. Only five patients had completed pulmonary function tests preoperatively because the others were unfit to undergo them due to complications on admission, such as pneumothorax, or noncompliance.

Postoperatively 12 patients were classified as having Group I bullous disease, and eight with Group II bullous disease. The bullae were located on the right side in 12 patients, on the left side in seven patients, and bilaterally in one patient (Table 1). Out of 21 GLBs, eight had a wide attachment with lung parenchyma (wide-based bullae's) and 13 had a short attachment (short-based bullae's). Fifteen patients (75%) had a GLB with related symptoms (dyspnea, chest pain) or complications on admission (infection, pneumothorax). Five patients (25%) were operated electively due to an incidental finding of a GLB occupied more than one-third of the hemithorax in all of them, and in one an additional incidental solitary pulmonary nodule adjacent to the bullae was diagnosed on postoperative pathologic examination. This patient underwent completion lobectomy upon confirmation of lung cancer two weeks later. One patient (5%) with bilateral GLB underwent a staged bilateral VATS bullectomy. He had initially undergone left side surgery because of a secondary spontaneous pneumothorax (SSP) and elective right side surgery three months later.

SSP was the predominant presenting complaint in nine out of 20 study patients (45%), including two patients with tension pneumothorax, four patients with non-resolving pneumothorax, and three patients with recurrent pneumothorax. Breathlessness (without SSP) was the initial symptom in five patients (25%). Two patients (10%) were admitted with infected bullae, and a surgical resection was performed after initial antibiotic therapy and only after resolution of the acute infection. A CT scan confirmed the diagnosis preoperatively in all patients. GLB were more common in the upper lobes (15 patients, 75%), and a mediastinal shift was seen in three patients (15%).

Eight out of 21 surgeries (38.1%) were thoracotomies and 13 out of 21 surgeries (61.9%) were VATS bullectomies. There was no intraoperative or hospital mortality and conversion to thoracotomy was carried out in one out of 14 VATS surgeries because of severe adhesions. The operating time ranged from 30 to 155 min (median, 75 min). Adhesions were identified in 12 out of 21 surgeries (57%).

Complications were seen during 10 out of 21 surgeries (47.6%). Three patients (14.2%) had pneumonia, one patient (4.8%) had wound infection, and six patients (28.5%) had prolonged air leak of whom three (14.2%) developed empyema, two of them (9.6%) needed reoperation (both underwent decortication with closure of the leakage), three of the six patients with prolonged air leak were discharged with a Heimlich valve (Table 2). The median duration of hospitalization was nine days (range 3–34 days).

**Table 2:** Pathologies and management of the patients with prolonged air leak

Patient	Multiple bullae	Pleural adhesions	Empyema	Reoperation	Discharged with Heimlich valve	Hospital stay (days)
1	+	+	+	+	+	34
2	+	+	−	+	−	10
3	+	−	+	−	+	17
4	+	+	+	−	+	21
5	+	+	−	−	−	7
6	−	+	−	−	−	21

## Discussion

Surgical intervention is the last resort for treating giant bullae because of the wide variety of factors that affect the postoperative course and make the decision to operate highly challenging. Preoperative assessment is critical for patients with symptomatic or complicated bullous emphysema [13]. A CT of the chest can provide detailed information not only on location, size, and number of bullae, but also on adjacent pleural, mediastinal, and underlying pulmonary parenchima [8, 14]. It is vitally important to assess the patient's pulmonary function, including pulmonary ventilation volume-flow relationships, prior to embarking upon surgical intervention. Patients with ruptured bullous emphysema who sustain a persistent air leak are unfit to undergo this assessment, and other means, such as determining the dyspnea index, diffusion capacity of carbon monoxide, blood gas analysis, six-minute walking test, or pulmonary perfusion-ventilation radio-nucleotide scanning will need to be used [6, 8, 15, 16]. Despite the fact that these parameters are crucial to the selection of the most suitable surgical procedure, they are not performed generally due to the emergent nature of the surgery.

Several factors, such as a single giant bullae, a significantly compressed normal lung, and the absence of severe chronic obstructive pulmonary disease are suggested as important predictors of a good postoperative outcome [17]. The development of VATS in the 1990s has changed the timing and indications of surgical intervention for GLB because of its minimally invasive nature and the level of effectiveness equivalent to that of open procedures [18, 19].

The indications for bullectomy to prevent as well as to treat complications of bullae are well known [20, 21], but there has been no compelling evidence to favor one surgical technique over another. The most common indications for bullectomy are the prevention of recurrence, the treatment of a non-resolving pneumothorax, and the improvement of dyspnea. The prevention of pneumothorax largely depends upon whether or not there is only a single GLB—which is associated with normal lung parenchyma—or if the GLB as a part of emphysematous lung disease. In such cases, bullectomy can also be accomplished by mechanical pleurodesis.

At maximal inflation, bullae act as space-occupying regions that compress subjacent pulmonary tissue. Hyperinflation, dead-space ventilation, and increased work of breathing can also be observed. GLB may be viewed as “intrapulmonary pneumothorax”, meaning that if the underlying parenchyma is preserved, a bullectomy will lead to re-expansion of the healthy lung, demonstrating the same effect as drainage of a primary pneumothorax. Therefore, removal of a single GLB that has evidence of compression of relatively normal lung parenchyma is believed to achieve the best postoperative outcome. If the compressed lung is emphysematous, however, the expansion of these regions may result in only regional improvements in compliance and gas exchange, with little effect on overall mechanics [5].

Some authors have suggested that bullectomy should not be indicated in patients with underlying emphysema, because it may not be helpful in relieving dyspnea. On the other hand, there is some evidence that bullectomy may act as Lung Volume Reduction Surgery (LVRS) in the same manner and yield similar functional results in patients with end-stage emphysema. Although bullectomy and LVRS are considered as two different surgical procedures, both allow the removal of a redundant space-occupying destructive emphysematous lung, permitting better ventilation and perfusion, a decrease in dead space and residual volume, and an improvement in chest mechanics with repositioning of the diaphragm and thoracic wall. Mineo et al. hypothesized that the bigger the bullae, compared with residual volume, the greater the possible benefits, with good and long-lasting results for a bullae: residual volume ratio of more than 30% [22].

Gunnarsson et al. found that three-quarters out of 63 patients who underwent resection for GLB developed postoperative air leak (7). Zhu et al. found that patients aged > 48 years, smokers and patients with emphysema, are more likely to experience postoperative complications (11).

They reported 35.7% air leak (in ten patients) after resection of GLBs, and noticed that presence of emphysema were more prone to postoperative air leaks (52% vs. 15.8%, *p* = 0.013). We reported in our series a 24.5% air leak (6 out of 20 patients) with two re-surgeries in patients that had a wide-based GLBs. Resection of wide-based GLB requires a large-volume reduction on underlying pulmonary parenchyma and is technically more difficult and compared with the technique of LVRS. In such a cases the use of buttressing material should prevents postoperative persistent air leak.

Patients who have bullous lung disease in the presence of diffuse parenchymal involvement (emphysematous or non-emphysematous) should be evaluated on an individual basis, and surgery should be performed for those who would highly benefit from even a small increase in pulmonary function [1]. In a retrospective series by De Giacomo et al., VATS bullectomy for selected patients with emphysematous bullous disease resulted in similar pulmonary functional improvements when compared with a matched group of patients undergoing lung volume reduction [23]. Randomized trials of giant bullectomies have not yet been performed, but observations from numerous case series suggest that resection of giant bullae in carefully selected patients is associated with symptomatic and functional improvements lasting for five or more years in 60–90% of the patients [1, 10, 11, 14, 16].

### Limitation

This series of 20 patients who underwent bullectomy due to giant bullae with acceptable morbidity and complication rates are limited by the lack of data on the patients’ postoperative functional status during the 24-month follow-up period. For example, the patient after staged VATS bullectomy improved significantly postoperatively and his pulmonary function test results revealed the increased Forced Expiratory Volume in 1 s (FEV1) to 86% compared to 60% preoperatively (FEV1 3.65L compared to 2.6 L). All the other patients were satisfied with the surgical outcome and complied with the outpatient observation protocol.

## Conclusions

Surgery is the best treatment option for giant lung bullaes, and our experience has provided evidence that it can be performed safely by VATS of thoracotomy with acceptable postoperative outcomes. Minimally invasive surgery as an open thoracotomy surgery is safe and effective for giant lung bullae, and in our opinion, VATS should be considered the first-line approach technique. Patients with wide-based GLBs are more likely develop postoperative complications to compare with short-based GLBs patients.

## Data Availability

The datasets used and/or analyzed during the current study are available from the corresponding author on reasonable request.

## Abbreviations

VATS: Video-assisted thoracoscopic surgery
CT: Computerized tomography
SSP: Secondary spontaneous pneumothorax
LVRS: Lung volume reduction surgery
FEV1: Forced expiratory volume in 1 second
